# Lessons From a Near-Peer Junior Doctor Teaching Program in Trauma and Orthopedics

**DOI:** 10.7759/cureus.31788

**Published:** 2022-11-22

**Authors:** Fitzgerald Anazor, Nachappa Sivanesan Uthraraj, Nik I Bakti

**Affiliations:** 1 Trauma and Orthopaedics, William Harvey Hospital, Ashford, GBR

**Keywords:** clinical teaching, medical education, near-peer teaching, teaching feedback, trauma and orthopaedics

## Abstract

Introduction: A junior doctor teaching program delivered by near-peers can foster collaboration in a less-pressured and conducive learning environment. The aim of this study was to provide an analysis of an orthopedic teaching program in a high-resource environment utilizing readily available tools and resources that are potentially available in most hospitals globally.

Methods: This study involved the utilization of an outcome-based learning approach with regular formal feedback. An anonymized Google Forms survey using a 10-point Likert scale was conducted after a 30-week period. The survey tool was sent out to 28 doctors and two senior nurse practitioners who participated in the program either as tutors, learners, or both. A total of 19 out of 30 respondents completed the survey giving a 63% survey completion rate. The setting for this study was the trauma and orthopedics department in a United Kingdom district general hospital.

Results: Learners’ confidence in their orthopedic knowledge and skills pre-program had a median response of eight with a mode of seven whereas confidence following engagement on the program improved with a median response of nine and a mode of 10. At an alpha level of 0.05, this observed improvement was statistically significant using the Mann-Whitney U test (p=0.034). Tutors’ perception of the usefulness of the teaching feedback had a median response of nine with a mode of 10. Relevance of the selected topics had a median response of nine and a mode of 10. Inclusion in the teaching program to cater to learner diversity had a median response of nine and a mode of 10. The effectiveness of a blended approach for learning had a median response of nine and a mode of 10.

Conclusion: This study has provided evidence of the benefits of a near-peer teaching program. This is especially important in the post-coronavirus disease (COVID) pandemic recovery period where easily accessible and well-grounded educational programs will be useful to complement the deanery teachings for trainees. This is important as this may be the main source of formal teaching for non-trainee junior doctors in many hospital settings. Additional research will be needed to further explore the pros and cons of such programs within a surgical specialty like orthopedics with an emphasis on the various pedagogical approaches to teaching and learning for junior doctors working in a busy clinical setting.

## Introduction

Near-peer teaching can be described as teaching by tutors who are a few years ahead of learners but on the same training or career spectrum [1]. A junior doctor in the setting of this study refers to any doctor who has completed foundation year one training (equivalent to the one-year internship/house officer role in some other countries) but is below the level of a consultant. A registrar is a junior doctor who has completed core training or junior residency training while a senior house officer or core trainee is a junior doctor who has completed foundation training/internship. There is literature evidence on the benefits of near-peer tutors in delivering teaching sessions to medical students and junior doctors with benefits including collaborative working, relatively inexpensive tutors compared to full-time lecturers, and the ability of the tutors to tailor the sessions to suit the learners due to similar shared experiences. Furthermore, other benefits include the fact that junior doctor colleagues are likely to be more approachable compared to more senior trainers, the improved confidence for tutors, and finally, tutors demonstrating improved knowledge of taught topics [2-6]. 

There are challenges with conducting teaching sessions for doctors in clinical practice, especially with the current pressures faced by the United Kingdom (UK) National Health Service (NHS) as a result of the COVID-19 pandemic-induced backlog. This means that such teaching programs have to strike a delicate balance between the clinical workload and teaching. A junior doctor teaching program delivered in a structured manner with appropriate educational principles and quality feedback has the potential to contribute to improved patient care delivery through the provision of important knowledge and skills to junior doctors. The correlation of knowledge gained from theoretical discussions to real-life clinical practice is quite beneficial in an experiential learning model from the concrete shared experience to active experimenting as part of Kolb's learning cycle [7-8]. Medical education in this format is also an important component of continuous professional development and clinical governance. The UK General Medical Council (GMC) outlines the importance of doctors maintaining and developing their teaching skills as part of good medical practice [9]. 

The Joint Committee on Surgical Training (JCST) in the United Kingdom recommends a minimum of two hours of a formal teaching program per week for all trainees [10]. The teaching program thus established in our department serves to provide part of this in addition to the weekly deanery teaching available to trainees. Furthermore, it serves as the main source of formal teaching for junior doctors in non-training posts who play a significant role in patient care delivery. 

The aim of this study is to present the learning points gained from this teaching program and provide a critical analysis of the strengths, weaknesses, opportunities, and possible threats (SWOT) facing such programs, in order to guide other program designers who are currently implementing or plan to implement similar programs within their various local contexts. 

## Materials and methods

The setting for this study was an NHS district general hospital or trauma unit in the southeast of England. We instituted a junior doctor teaching program targeted at trauma and orthopedics core trainees, foundation year doctors, trust grade house officers, and rotating medical students within the department. Senior trauma nurse practitioners also participated as learners in the program. Tutors were mainly registrar-level doctors (specialty trainee level three equivalent and above) while some sessions involving orthogeriatric topics were delivered by the orthogeriatric consultants. Consultants were in attendance to provide supervision for 11 out of 30 sessions (37%). 

Sessions were delivered once weekly on a fixed day and time. Each session was designed to last approximately 45 min. A structured teaching format based on an outcome-based learning approach was utilized. A list of orthopedic and orthogeriatric topics selected based on feedback from the junior doctors and the tutors was fed into a rota schedule displaying the dates and names of tutors (Table 1). This was published in late October prior to the commencement of the program. Thus, the tutors and learners had enough time to prepare for each session. There was no bleep-free/protected time allocated for this program due to rota pressures as a result of the high clinical workload and clinicians demonstrated some flexibility in order to combine the program with their clinical work. However, the sessions were delivered in the afternoons between 13.00 and 14.00 Greenwich Mean Time (GMT) when there was an anticipated slightly lower clinical work load.

**Table 1:** List of topics delivered per teaching session during the 30-week period. VTE, venous thromboembolism; DNACPR, do not attempt cardiopulmonary resuscitation; TEP, treatment escalation plan; ATLS, advanced trauma life support

Topics taught on the program
Interpreting plain radiographs in orthopedics
Appropriate clinical presentation at trauma meetings -- a focused approach
Initial management of patients with femoral neck fractures on admission
Management of limb compartment syndrome
Hyponatremia in the orthopedic patient
VTE prophylaxis in the orthopedic patient
Initial care of the critically ill orthopedic patient in the ward
Closed reduction of common joint dislocations -- shoulder, elbow, hip, ankle
Principles of orthopedic manipulation and casting-distal radius fracture as a typical example
Acute kidney injury in the orthopedic patient
How to handle difficult interactions with colleagues while on call/on the ward
Initial management of pediatric supracondylar fractures
Management of the acutely painful large joint in an adult with suspicion for septic arthritis
Initial assessment of the limping child
Clinical evaluation and decision-making while on call for a patient with suspected cauda equina syndrome
Management principles in a patient with an open fracture
DNACPR and TEP-current best practice
Basic principles of resuscitation and initial management of the trauma patient
Use of fluids and fluid/electrolyte balance in the orthopedic patient
Management of shoulder dislocations
Trauma calls -- ATLS principles
Principles of orthopedic fracture manipulation and casting
Management of the diabetic orthopedic patient going for surgery
Carpal tunnel syndrome -- clinical assessment and treatment
Metabolic response to trauma/surgery -- clinical implications
Initial management of hip dislocations
Principles of management of ankle fractures
Principles of management of pediatric physeal injuries
Best practice tariff for hip fractures -- how can we improve at junior doctor level?
How to handle difficult interactions with patients

Peer collaborative discussions and review workshops were used for most sessions. Sessions were delivered in a blended manner to promote access and flexibility with a combination of face-to-face and virtual attendance using Webex (Cisco Systems, CA, USA). Formal feedback was collected using the Survey Monkey online tool (Momentive, NY, USA) for each session and used to improve future sessions. The attendance for each session ranged from 5 to 15 doctors over the duration of the program. There was no summative assessment and attendance was voluntary but encouraged. Formative assessments in the form of clinical problem-based scenarios and interactive multiple-choice questions were built into the sessions utilizing "buzz groups" of two to three students per group.

The program was analyzed after a 30-week period (November 3, 2021-May 31, 2022). This analysis was conducted using anonymized responses collated via online Google Forms (Google, CA, USA) sent out at the end of the 30-week period. The survey tool was sent out to 28 doctors and two nurse practitioners who participated in the program either as tutors, learners, or both. Feedback responses were provided on a 10-point Likert scale with one being the lowest rating for the assessed item in the questionnaire and 10 being the highest rating or best available positive response (see Appendix section). Data were transferred to a Microsoft Excel sheet (Microsoft Corporation, CA, USA) for analysis.

## Results

Overall, a total of 19 respondents completed the survey giving a 63% survey completion rate. Some 57.9% of respondents were learners in the program, 10.5% served as tutors while 31.6% were both tutors and learners on different occasions. Some 94.7% of respondents were working in orthopedics while 5.3% were in orthogeriatrics. Some 31.6% of respondents were registrars, 26.3% were resident medical officers, 15.8% were trust grade senior house officers, 15.8% were core trainees, and 10.5% were orthopedic nurse practitioners.

**Figure 1:**
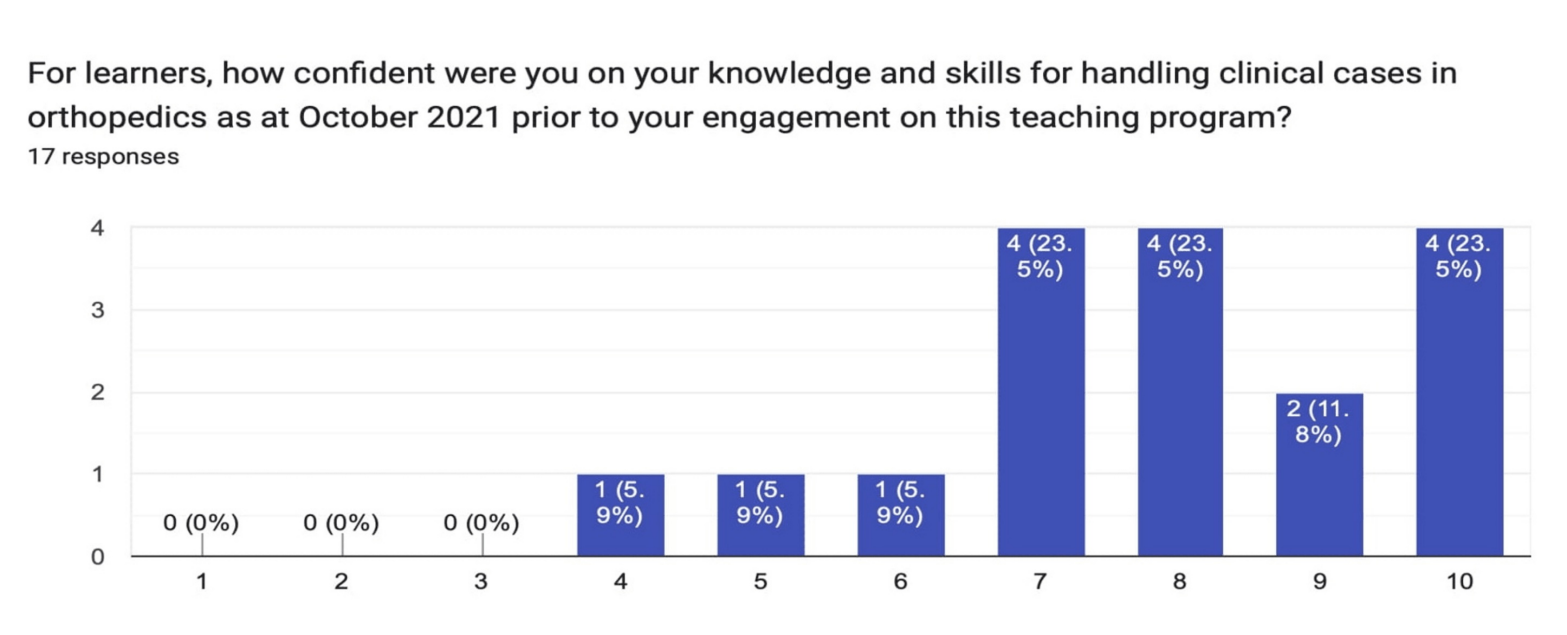
Chart summarizing the distribution of the responses obtained from learners gauging their orthopedic knowledge and skills pre-program participation.

For respondents' confidence in their orthopedic knowledge and skills prior to the commencement of this program, out of the 18 completed responses for this domain, the median response was eight, and the mode was seven (Figure 1).

**Figure 2:**
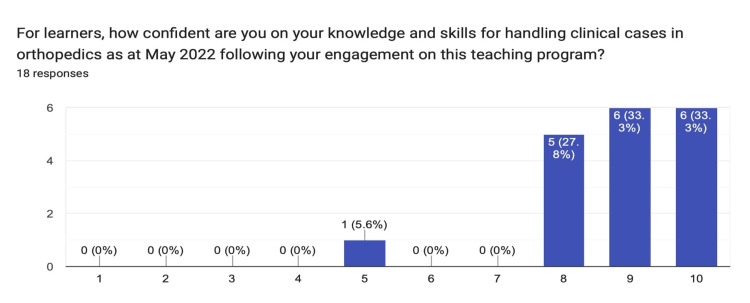
Chart summarizing the distribution of the responses obtained from learners gauging their orthopedic knowledge and skills post program participation.

The confidence in their orthopedic knowledge and skills following active engagement in the program provided a median response of nine and a mode of 10 (Figure 2).

**Figure 3:**
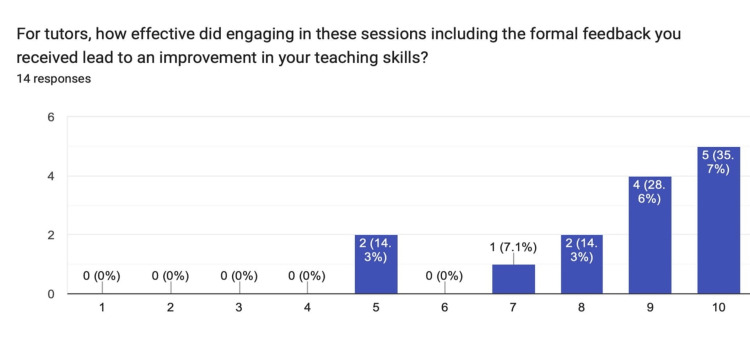
Chart summarizing the responses from the tutors regarding the usefulness of the formal feedback provided per teaching session.

This observed improvement in clinical knowledge and skills was statistically significant at an alpha level of 0.05 using the Mann-Whitney U test (p=0.034). For tutors’ perception of the usefulness of the formal feedback provided per teaching session, the median response was nine with a mode of 10 (Figure 3).

**Figure 4:**
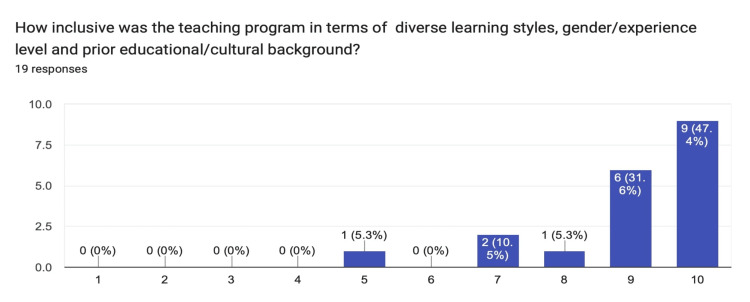
Chart summarizing the responses from the tutors and learners for program inclusion to cater to diversity.

Relevance of the selected teaching topics among both tutors and learners had a median response of nine and a mode of 10. The inclusion of the teaching program to cater to learner diversity had a median response of nine and a mode of 10 (Figure 4).

**Figure 5:**
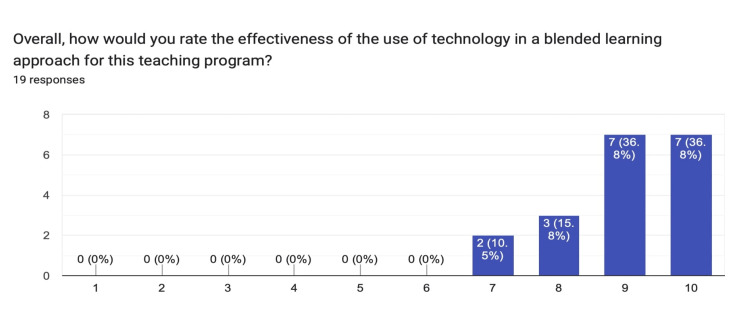
Chart summarizing the respondents' ratings for the use of technology for delivering a blended learning approach.

For the effectiveness of the use of technology to improve access to sessions via a blended approach, the median response was nine and the mode was 10 (Figure 5). 

Some 89.5% of respondents felt the presence of a consultant to provide direct supervision for each session would have been beneficial while 79% felt a bleep-free or protected time period for the once-weekly teaching program was needed. 

## Discussion

As seen from the results, this teaching program increased learners' confidence in their orthopedic knowledge and skills. A randomized control trial employing near-peer teaching on a surgical skills course demonstrated improved learner performance in some skill-set domains [11]. However, one might argue that having confidence does not translate to actually transferring the knowledge and skills learned. This is probably countered by the earlier-mentioned fact that experiential learning was a key component for outcome-based learning sessions. In addition, employing an outcome-based learning approach provides progression to the critical and reflective spectrum of clinical teaching and learning through aligning the learning session and formative assessments to take into account learners' background knowledge and preconceptions [12-13]. This deeper approach to learning is probably better than simply learning through "transmission" which might be preferred by some learners especially those accustomed to this style [14]. Orthopedics is a specialty that involves a lot of practical skills and thus, active learning with reflective practice is key to delivering quality education. 

For this study, the learners were quite diverse in terms of medical training with some being UK-trained and others international medical graduates. There was some variation amongst learners in terms of knowledge level, skill level, cultural differences, being a trainee vs non-trainee, gender, ethnicity, and work commitments. The program had to be inclusive bearing in mind this rich learner and tutor diversity, in order to address both intrinsic and extrinsic factors that could potentially affect learning. A learning environment as obtained in this teaching program allows for open interaction and questioning fostering psychological safety [15]. Learner diversity feeds into the concept of internationalization in medical education with the NHS having a diverse workforce with a lot of foreign-trained clinicians in the setting of learning transcending international boundaries, especially following the COVID-19 pandemic [16]. As seen in the results from this study, most respondents rated the program highly on inclusion. The topics were also diverse and relevant ranging from common orthopedic to orthogeriatric clinical and professional problems or scenarios. 

Another strength of the program was the well-defined structure and ease of access as it was delivered through a blended learning approach enabling clinicians who were working off-site to participate. This was reflected in the positive response from most learners and tutors in the program. 

Finally, the results from this study showed that most respondents found the use of formal feedback to be important and beneficial. There was a strong utilization of reflective practice through formal feedback per session and utilized to improve future sessions using Pendleton’s model [17]. The program also employed peer feedback through peer-review discussions, consultant supervisor feedback through observation of teaching (OOT), and multi-source feedback (MSF). Both tutors and learners had a rich array of quality feedback to reflect upon. The feedback conformed to most of the principles of effective feedback including appropriate structure, effective collaboration between tutors and learners, positive motivational beliefs, and closing the gap between current and desired practice [18]. 

Some of the limitations of this study include the lack of a bleep-free time period for these sessions which was due to the clinical pressures meaning some clinicians had to employ a great deal of flexibility and time management to actively engage with the teaching sessions. This was the main limitation identified in the survey of learners and tutors on the program. A potential way of overcoming this challenge might be to allocate a single bleep holder for the wards during the teaching program except when an emergency situation arises. This technique is utilized by some other NHS hospitals but this is a relatively complex issue to address. Another possible limitation is the lack of consultant supervision for all sessions. This may not be a major issue if anybody implementing a similar program ensures it is well-structured and built on a sound medical education pedagogy. Furthermore, orthopedics has a good culture of daily trauma meetings which provides an avenue for consultant teaching in most cases. However, consultant attendance to provide supervision at the formal teaching program potentially helps foster teamwork and provides an avenue for junior doctors to gain clarifications on certain complex issues beyond the time-constrained trauma meeting setting. Another limitation of this study is the lack of a control group in order to compare the results. Finally, a larger sample size could have increased the study quality. However, the population studied was restricted to a single orthopedic unit in a single hospital. 

Opportunities for future development of the index program or similar programs in other hospital settings exist. Formal training/courses on higher education learning theory and practice can be provided for all tutors within such a program, in order to improve the learning experience. Ring-fenced sources for funding for practical sessions on such programs will be useful. This can be provided through appropriate collaboration with the industry or through utilizing the department’s medical education budget. A certificate or letter of recognition for teaching in such programs can also improve enthusiasm and be useful evidence for annual appraisal or review of competence progression. Anybody planning a similar program should be aware of the aforementioned challenges and threats to implementing a successful program. In addition, there has to be an effective, highly motivated leader of the program for it to be maintained and successful. Effective collaboration with colleagues and the clinical leadership of the department should be cultivated in order to ensure such a program is well-institutionalized. Regular audits of such programs as part of quality improvement will ensure standards are maintained and innovations are introduced on a regular basis.

## Conclusions

This study has demonstrated key lessons for a junior doctor teaching program in orthopedics employing near-peers for teaching. This has not been extensively studied in the literature as it applies to trauma and orthopedics. An analysis of the program has been provided to guide other medical educators planning or already engaged in similar programs. Such a program can complement the deanery-organized teaching for trainees and maybe the main source of regular, formal teaching for non-trainee junior doctors. 

This study has provided evidence within a relatively small cohort of the benefits of such a teaching program, especially in the post-COVID pandemic recovery period where easily accessible, cost-effective, and well-grounded educational programs will be useful for many hospitals and departments. These can be adapted for use even in low-to-middle-income settings. Additional research will be needed to further explore the strengths and limitations of such programs within a surgical specialty like orthopedics with an emphasis on the various pedagogical approaches to teaching and learning for junior doctors working in a busy clinical setting.

## Figures and Tables

**Figure 6:**
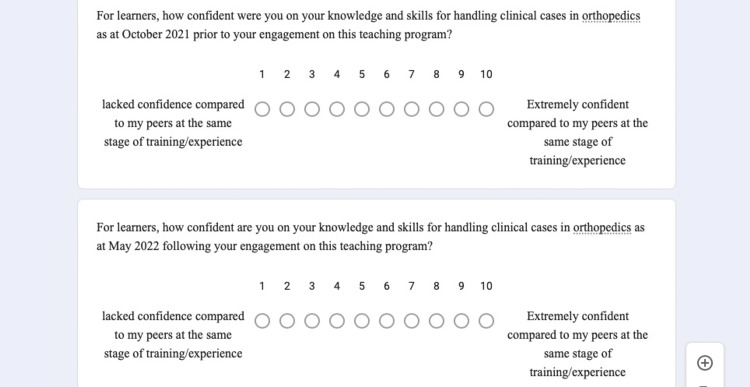
Sample section of the questionnaire utilized for the study showing the 10-point Likert scale.
