# Correction: Lessons From a Near-Peer Junior Doctor Teaching Program in Trauma and Orthopedics

**DOI:** 10.7759/cureus.c250

**Published:** 2025-08-19

**Authors:** Fitzgerald Anazor, Nachappa Sivanesan Uthraraj, Nik I Bakti

**Affiliations:** 1 Trauma and Orthopaedics, William Harvey Hospital, Ashford, GBR

This article has been corrected as follows. The Mann-Whitney U test p-value was incorrectly stated as 0.466 in the abstract and results section. This has been corrected to 0.034.

